# Oral Surgery and Osteoradionecrosis in Patients Undergoing Head and Neck Radiation Therapy: An Update of the Current Literature

**DOI:** 10.3390/biomedicines11123339

**Published:** 2023-12-18

**Authors:** Giulia Corrao, Giovanni Carlo Mazzola, Niccolò Lombardi, Giulia Marvaso, Alberto Pispero, Elisa Baruzzi, Sem Decani, Marco Tarozzi, Luca Bergamaschi, Chiara Lorubbio, Ilaria Repetti, Anna Starzyńska, Daniela Alterio, Mohseen Ansarin, Roberto Orecchia, Fiorella D’Amore, Roberto Franchini, Andrea Nicali, Paolo Castellarin, Andrea Sardella, Giovanni Lodi, Elena Maria Varoni, Barbara Alicja Jereczek-Fossa

**Affiliations:** 1 Division of Radiation Oncology, IEO—European Institute of Oncology, IRCCS, 20141 Milan, Italy; giulia.corrao@ieo.it (G.C.); giovannicarlo.mazzola@ieo.it (G.C.M.); giulia.marvaso@ieo.it (G.M.); luca.bergamaschi@ieo.it (L.B.); chiara.lorubbio@ieo.it (C.L.); ilaria.repetti@ieo.it (I.R.); daniela.alterio@ieo.it (D.A.); barbara.jereczek@ieo.it (B.A.J.-F.); 2 Dipartimento di Scienze Biomediche Chirurgiche e Odontoiatriche, Università degli Studi di Milano, Via Beldiletto 1, 20142 Milan, Italy; niccolo.lombardi@unimi.it (N.L.); alberto.pispero@unimi.it (A.P.); elisa.baruzzi@unimi.it (E.B.); sem.decani@unimi.it (S.D.); marco.tarozzi@unimi.it (M.T.); fiorella.damore@unimi.it (F.D.); roberto.franchini@unimi.it (R.F.); andrea.nicali@unimi.it (A.N.); paolo.castellarin@unimi.it (P.C.); andrea.sardella@unimi.it (A.S.); giovanni.lodi@unimi.it (G.L.); 3 ASST Santi Paolo e Carlo, SC Odontostomatology II, San Paolo Hospital, 20142, Milan, Italy; 4 Department of Oncology and Hemato-Oncology, University of Milan, 20122 Milan, Italy; 5 Department of Oral Surgery, Medical University of Gdańsk, 7 Dębinki Street, 80-211 Gdańsk, Poland; anna.starzynska@gumed.edu.pl; 6 Division of Otolaryngology and Head and Neck Surgery, IEO European Institute of Oncology, IRCCS, 20141 Milan, Italy; mohseen.ansarin@ieo.it; 7 Scientific Directorate, IEO-European Institute of Oncology, IRCCS, 20141 Milan, Italy; roberto.orecchia@ieo.it

**Keywords:** head and neck radiotherapy, head and neck cancer, osteoradionecrosis, oral surgery, oral medicine, IMRT

## Abstract

Osteoradionecrosis (ORN) is a serious long-term complication of head and neck radiotherapy (RT), which is often triggered by dental extractions. It results from avascular aseptic necrosis due to irradiated bone damage. ORN is challenging to treat and can lead to severe complications. Furthermore, ORN causes pain and distress, significantly reducing the patient’s quality of life. There is currently no established preventive strategy. This narrative review aims to provide an update for the clinicians on the risk of ORN associated with oral surgery in head and neck RT patients, with a focus on the timing suitable for the oral surgery and possible ORN preventive treatments. An electronic search of articles was performed by consulting the PubMed database. Intervention and observational studies were included. A multidisciplinary approach to the patient is highly recommended to mitigate the risk of RT complications. A dental visit before commencing RT is highly advised to minimize the need for future dental extractions after irradiation, and thus the risk of ORN. Post-RT preventive strategies, in case of dento-alveolar surgery, have been proposed and include antibiotics, hyperbaric oxygen (HBO), and the combined use of pentoxifylline and tocopherol (“PENTO protocol”), but currently there is a lack of established standards of care. Some limitations in the use of HBO involve the low availability of HBO facilities, its high costs, and specific clinical contraindications; the PENTO protocol, on the other hand, although promising, lacks clinical trials to support its efficacy. Due to the enduring risk of ORN, removable prostheses are preferable to dental implants in these patients, as there is no consensus on the appropriate timing for their safe placement. Overall, established standards of care and high-quality evidence are lacking concerning both preventive strategies for ORN as well as the timing of the dental surgery. There is an urgent need to improve research for more efficacious clinical decision making.

## 1. Introduction

In the European Union, 18 out of every 100,000 inhabitants are diagnosed with head and neck cancer (HNC) each year, with men accounting for the majority of cases, while worldwide, around 900,000 new cases are identified yearly [1,2].

HNC includes a heterogeneous group of tumors, in which smoking and alcohol consumption have historically been the most important etiological risk factors [3], although today, human papillomavirus (HPV) infection is responsible for about three-quarters of all oropharyngeal cancers [4].

Surgery, radiation therapy (RT), and chemotherapy, according to the tumor stage, are the main treatments used for HNC. Specifically, according to the type of tumor and localization, early-stage tumors can be successfully treated with surgery or RT alone, while locally advanced disease requires multimodal treatments, including surgery followed by adjuvant RT with or without concomitant chemotherapy [5].

RT involving the oral cavity carries several possible acute adverse effects, which are more likely when combined with concomitant chemotherapy, including mucositis, xerostomia, dysphagia, pain, erythema, dysgeusia, and long-term toxicity of the masticatory muscles, teeth, and bones [6].

Intensity-modulated RT (IMRT) is the currently preferred procedure for HNC and is now considered a standard of care for treating HNC [7]. Compared to three-dimensional conformal RT (3DCRT) and surgery, IMRT allows enhanced organ and functional preservation by decreasing the incidence of side effects [8].

This narrative review aims at reporting a contemporary analysis of the osteoradionecrosis (ORN) risk in head and neck RT patients, who undergo oral surgery, with a focus on the timeframe for oral surgery and exploring preventive measures.

As known, ORN is defined as exposed irradiated jawbone not healing in 3 months, excluding any cancer-related local recurrence [9].

Ranging in incidence from 2 to 22% [10,11], ORN symptoms include pain, suppuration, and mucosal ulceration, along with bone necrosis and further bone exposure. In most severe cases, ORN can result in oral fistulae, infection, and pathological fractures of maxillofacial bones [12].

Overall, ORN occurs mainly in elderly patients, smokers, and habitual alcohol users with poor oral health and nutritional deficiencies, who received more than 60 Gy [13]. Comorbidities, such as diabetes mellitus and collagen vascular diseases, are additional risk factors [14,15]. Triggering variables include active periodontal disease and dental extractions, mainly localized at the mandibular alveolar bone [16]. The presence of hypoxia, hypocellularity, and hypovascularity in irradiated bone tissues represents the basis of ORN pathogenetic mechanisms, occurring after dental surgery [17]. A recent theory refers to the occurrence of progressive and long-lasting radiation-induced fibrosis (RIF) and atrophy [18,19].

Although the improvements in RT techniques (particularly the use of IMRT) showed a reduction of close to 10% in the incidence of ORN cases [20], this adverse event still represents a challenge for radiation oncologists and dentists, mainly due to the paucity of prospective data and inconsistencies of prevention, management, and follow-up protocols [21]. To date, proton therapy has been proposed for a more targeted treatment, but still appears associated with the risk of ORN [22,23].

The management of ORN does not have a gold standard approach, and it is highly variable, ranging from conservative management, including prescription of mouthwash, analgesics, antibiotics, and anti-fibrotic and anti-oxidant agents, to radical surgical strategies and/or hyperbaric oxygen therapy (HBO), according to the severity of the clinical picture [24]. Preventive strategies are, thus, strontly recommended [25] and include pre-RT dental visits to identify hopeless or doubted teeth to be removed. The final objective is to reduce the need for dental extractions post-RT, which can be regarded as the main triggering factor for ORN [26].

Overall, the literature is scarce on both the prevention and the management of ORN that are still based on low-quality observational studies. The ORN risk related to the different timing of when the dental extractions are performed has been under debate for a long time, and there is still no consensus. Other open questions are related to the medical preventive strategies (e.g., HBO or pentoxifylline and tocopherol) able to decrease the risk of ORN after tooth removal and dental implant placement in RT patients. Today, a general consensus on the effective strategies to prevent this rick, in the light of well-designed and multicenter studies, remains an urgent need.

This review aims at providing an update of the current literature, shedding light on the suitable timing for oral surgery (dental extraction or implant placement) and medical preventive treatments for patients, who received head and neck RT, to decrease the risk of ORN.

## 2. Methods

This paper is a narrative review focusing on relevant investigations in the prevention of ORN.

A search was performed in PubMed through July 2023 with the following keywords and synonyms: “Timing dental extraction radiotherapy”, “Timing dental implant radiotherapy”, “Osteoradionecrosis prevention pentoxyphylline tocopherol”, and “Osteoradionecrosis prevention hyperbaric oxygen”. Keywords and index/subject terms were joined by Boolean operators “AND” or “OR”. PubMed was used as a unique database since the main aim of this narrative review was to retrieve updated clinical evidence, published on the topic.

Studies were included only if patients were 18 years and older and if publication was available in full and in English. Interventions, clinical trials, observational studies, and systematic reviews were also considered. Articles published before 2010, not in English, not concerning HNC, abstracts, commentaries, and editorials were excluded.

The results were further selected by the authors according to study type, citation count, year of publication, and English language, and they were then utilized as the basis for this review’s references. Reviewing citation listings and manually checking pertinent reference lists for articles missed by the primary search were examples of secondary searching.

## 3. Results and Discussion

The timing of oral surgery, particularly tooth extractions, can influence the risk of developing ORN in irradiated HNC patients, and it is an important aspect to be considered for the correct management of the patient. Beyond this aspect, there are some preventive strategies that have been suggested to reduce the risk related to ORN in the already irradiated patient, as detailed below.

### 3.1. Optimal Timing for Dento-Alveolar Surgery in Cases of Head and Neck RT

#### 3.1.1. Dental Extractions

Dental extractions before the beginning of RT

Recent oncological guidelines recommend a preventive dental visit before starting RT, and suggest performing dental extractions of hopeless or doubted teeth or other surgical procedures at least 7–14 days before the beginning of RT [27,28]. Within one week from the tooth extraction, the blood clot fills the alveolar socket, and then it is replaced by the granulation tissue. The re-epithelialization, starting within 24 h from the intervention, ends after one or five weeks, according to the clinical picture [29]. Notably, the healing time can vary in each patient, depending for example on comorbidities, and site of extraction [30]. Dental extractions can be performed on (or before) the day of oncological resection, reducing the delay in RT starting without a significant impact on operating room time [31].

Although dental extractions before RT may also represent a risk factor for ORN and should be balanced with possible delays in starting cancer therapy [32], most available literature data recommend dental extractions prior to RT [33,34]. Most of the recent systematic reviews agreed on the usefulness of performing dental extractions before starting RT to reduce complications [32,35], together with the need to maintain a high level of oral health [36]. ORN risk is significantly lower than performing the dental extractions after or during RT [32,35,37]. Not all studies reported the time interval between the extractions and the beginning of RT, but, when mentioned, there was wide heterogeneity [32,35], ranging from 3 to 210 days with a median of 24.7 days [35]. On the other hand, a systematic review by Beaumont et al. reported that, in the studies examining pre-RT extractions, the pooled incidence of ORN in patients having extractions preceding RT was 5.5%, ranging from 0 to 9.3% [37]. However, the high heterogeneity in study designs and several important patient-related variables emphasized the importance of considering these results with caution.

Two clinical audits [38,39] retrospectively assessed the ORN occurrence in HNC patients receiving RT. They followed the guidelines of the Royal College of Surgeons of England, which recommend a pre-RT dental visit and timing for dental extractions of at least 10 days before the start of RT. King et al. reported that 47 patients received extractions with a median of 13 days before RT starting, and no patients were diagnosed with ORN [39]. Ward et al. evaluated a similar HNC population, with 102 patients requiring dental extractions before RT; in two cases (1.3%), patients developed ORN after RT, although the authors did not identify a clear correlation with the timing of pre-RT dental extractions [38].

Several retrospective observational studies have been conducted in the past decades, reporting controversial data. See Toh and colleagues reported six cases (out of 231 patients; 2.6%) of ORN post-IMRT; four of them occurred in sites of pre-RT dental extractions, from 1 up to 24 months after RT (median of 5 months) [40]. Interestingly, a slightly higher incidence of ORN was observed in the case of more than four extracted teeth and current smokers [40]. Other previous observational cross-sectional studies, which referred mainly to 3DCRT and involved very small sample sizes, showed a higher rate of ORN occurrence in patients who received tooth extraction before RT (50–75%) [41,42,43], and the time intervals between tooth extraction and the start of RT ranged from 13 to 19 days [42].

Overall, recent systematic reviews showed the usefulness of performing dental extractions before starting RT to reduce the risk of ORN [32,35], although high-quality studies are needed to evaluate the best time interval from RT to oral surgery.

Supplementary Materials Table S1 reports a summary of the main studies available in the literature.

Dental extractions during RT

Costa-Normando and colleagues identified five studies in their systematic review that described dental extractions during RT, considering 1265 patients, and the main recommendation was to perform dental extractions before the beginning of RT [35]. In the clinical audit by Ward et al., a patient refused dental extractions before RT, which were then performed when the patient developed pain during the RT treatment, with consequent ORN development [38]. On the other hand, Wanifuchi et al., focusing on prophylactic tooth extractions, reported only one patient who received dental extraction during RT without developing ORN [42].

Findings about dental extractions during RT are poor and still controversial; therefore, they should not be preferred over pre-RT dental extractions.

Dental extractions after RT

Recent systematic reviews confirm that tooth extractions performed after RT are related to a higher occurrence of ORN compared to extractions performed before the starting of RT [32,35]. However, available data are heterogeneous, and a consensus about a differential risk according to different post-RT timing for dental extractions has not been achieved.

Costa Normando and colleagues showed a significant association between post-RT extraction and ORN risk (OR: 2.02; 95% CI: 1.02–3.98). The authors reported ORN occurrence post-RT extraction ranging from 3 months to 13.4 years [35]. Conversely, a previous systematic review could not find a statistically significant difference between performing dental extraction before or after RT. The pooled incidence of post-RT ORN was 5.3% (95% CI, 2.9–8.2%) versus pre-RT ORN of 5.5% (95% CI, 2.1–10.1%) [37].

A retrospective analysis of 231 patients found no cases of ORN among the 16 patients who received post-RT dental extractions [40], whilst Wanifuchi et al. reported that all patients (*n* = 6) who received tooth extraction after RT developed ORN (100%), and the median time interval between tooth extraction and the end of RT was 37.5 months (range: 27–120 months) [42]. Further estimates of ORN, related to post-RT extractions, ranged from 10% to 21% [26,43].

Overall, the literature supports a post-RT variable risk of ORN, and the question about the most suitable timespan between the end of RT and the dental extraction is still open. There is insufficient information to determine the overall risk of ORN, and the timing reported by different studies appears highly variable and often unclear [37].

In 1987, Marx and Johnson explained their radiobiological theory, with a gradual decrease in vascularization, while increasing fibrosis over the time after RT, and encouraged that dental surgical procedure after RT should be performed in a range from one up to six months from irradiation to minimize the risk of ORN [44]. Along these lines, dental extractions after RT might be carried out 5–6 months post-RT, before the onset of progressive tissue fibrosis and hypovascularization [45,46]. Kuo et al. also concluded that the risk of developing ORN is reduced if dental extractions are performed within six months after RT [46]. However, a “bimodal pattern” of trauma-induced ORN was also proposed, with a first peak of risk at 3–12 months and a second peak at 24–60 months after RT [44]. To confirm multi-modal pattern of trauma-induced ORN, Nabil and Samman reported incidence rates of 7.5%, 22.6%, and 17% within the first year, two–five years, and five years after RT, respectively [47]. The incidence of ORN has been also shown to rise gradually after the first-year post-RT, reaching the peak four years post-RT [48]. In 2007, Lye described that the peak incidence of delayed healing occurred in the second year after RT [49], but ORN occurrence was reported even 30 years after RT [26], confirming that the risk of ORN post-extraction is present even after many years of RT [30,48]. However, a recent retrospective analysis of medical records and orthopantomography showed that dental extractions carried out more than 5 years after RT correlated with a decrease in the probability of ORN post-extraction with OR = 0.06 (95% CI 0.01–0.25); albeit the risk was not eliminated [26].

Few studies described the surgical and medical protocols for performing post-RT dental extractions. See Toh et al. performed the dental extractions under pre-operative antibiotics (2 g amoxicillin or 600 mg clindamycin, in case of allergy to penicillin), but did not prescribe post-operative antibiotics, and tooth extractions were performed using dental elevators and forceps; any sharp bone was removed using rongeurs [40]. Khoo reported a positive association between the primary closure of sockets and the onset of ORN; the soft tissue manipulation and tight multiple sutures might be associated with vascular damage, hindering the correct healing process. The primary closure is, thus, not mandatory in these patients, particularly surgical procedures are required, such as flap raising [26].

Further risk factors of ORN have been identified. Lee et al. reported that lower jaw surgery was correlated to higher ORN risk (*p* = 0.001) [50]. The resection margins have been suggested as factors interfering with the vascularization and mucoperiosteum contributing to ORN [37]. Poor oral health, periodontitis, advanced tumor stage, chemotherapy, and a history of heavy smoking or alcohol abuse are other aspects that may increase individual risk [37,51,52]. In addition, ORN prevalence could be decreased by reducing the number of extracted teeth during the same intervention (the removal of less than five teeth has been suggested [46]), which is in line with previous findings that showed less morbidity in case of a single tooth extraction than in case of multiple extractions [11].

A summary of the main studies is reported in Supplementary Materials, Table S2.

#### 3.1.2. Implant Placement in Patients Receiving Head and Neck RT

Prosthetic rehabilitation of HNC patients is essential for oral functions, aesthetics, and social impact. In patients who received head and neck RT, dental rehabilitation should be performed, primarily using removable prostheses [53]. Implant placement should be reserved only for specific and selected cases, since the risk of ORN cannot be completely ruled out in the case of implant surgery. However, considering the anatomical and technical challenges of removable rehabilitation in oral cancer survivors, implant-supported prostheses compared with non-implant-supported prostheses were associated with better quality of life in both irradiated and non-irradiated patients [54].

The implant placement in the HNC patients who will receive RT can be “immediate”, when the implant insertion occurs before RT or during the ablative tumor surgery (primary placement), or it can occur after RT, regardless of the time interval from RT (“secondary placement”).

Primary placement aims at obtaining osseointegration before the onset of RT bone damage, avoiding the need for supplementary surgical procedures for oral rehabilitation; the positioning of implants can be greatly facilitated by the recent computer-guided implant surgery [55]. On the other hand, primary placement could interfere with or delay the cancer treatment, and it is not always feasible in hospital settings. When implants are placed before RT, the implant survival rates appear, overall, comparable with those of patients not receiving RT (89.6% versus 98.6%), and the radiation dose does not appear to correlate with the success of osseointegration [56]. Korfage et al. reported five cases of ORN, representing 5% of the patients who underwent RT after implant placement [57]. Although the literature is scarce on this topic, the presumed risk of implant-associated ORN has been related to the backscattering of radiation, which might result in an increased dose of radiation in the surrounding bone in front of and next to the implants, with a range of 10–21% [58].

The secondary placement has the advantage of a better assessment of the postsurgical anatomy and considers the patient’s oncological prognosis. When implants are placed after RT, systematic reviews suggest that implant survival is lower in radiation-treated bone than in the controls (not receiving RT) [59,60,61], although a recent retrospective study could not find significant differences [62]. The literature, particularly, supports that implant success is drastically reduced when the patient receives an RT dose higher than 55 Gy, and it is reduced even more for dosages over 70 Gy [60,63]. Although the specific literature relating to dental implants and ORN is limited, it is reasonable to presume that similar pathogenetic processes may reduce implant survival.

Decision-making guidelines for the implant placement, according to the received radiation dose, have been proposed by Anderson et al. [64]. In case of <50 Gy (low risk), only standard precautions should be applied; between 50 and 65 Gy (moderate risk), there is a marginal correlation with failing implants and implant placement should be considered with caution; between 65 and 74 Gy (relatively high risk), placement is not advised, unless associated with other precautions, such as HBO treatment, to improve the osseointegration; finally, in case of >75 Gy (up to 120 Gy) (high risk), implant placement is not advised, since implant failure and ORN risk are high.

Considering the receiving bone site, implant osseointegration was higher in the irradiated mandible than maxilla and higher in the irradiated native bone than the grafted bone [59,60].

Although more studies are needed due to a general lack of RCTs, a window between 6 and 18 months after RT to place dental implants has been proposed [64]; this timing balances the need for the patient’s recovery from RT acute adverse effects, the possibility of a “normal” healing of the bone tissue receiving the implant, and the putatively reduced risk of long-term chronic complications, including vascular damage and ORN, which need more time to develop and appear to worsen after 18 months [65]. Other authors reported similar “optimal” timings of 6–12 months following RT [64,66,67], or, similarly, ranging from 6 to 15 months after RT [55,68]. The placement of dental implants performed several years after RT is more at risk of ORN than early placement because of the decrease in bone healing potential [69]. Conversely, in 2013, a systematic review found a risk of failure that was associated with dental implants placed between 6 and 12 months post-RT of 34% higher than implants placed after more than 12 months from radiation [70]. A further retrospective study showed improved outcomes when the implant was placed at least 14 months post-RT, waiting at least six months for the loading [71].

As a general recommendation, after RT, considering the long-lasting risk of ORN and the lack of high-quality evidence on the best surgical timing, it is advisable to prefer an oral rehabilitation based on removable prosthesis as the first choice and consider the implant placement only in selected cases at the specific request of the patient for aesthetic-functional reasons and not otherwise effectively rehabilitated.

A summary of the main studies is reported in Table 1.

### 3.2. Preventive Strategies for Reducing the Risk of ORN Related to Dental Extractions

A recent systematic review [72] summarized the main approaches proposed in the literature to reduce the risk of ORN after dental extractions in patients who underwent head and neck RT. The review included both direct and indirect interventions to prevent the onset of ORN and found one single study [73] supporting the use of platelet-rich plasma after the removal of healthy teeth prior to starting RT; another study [74] found no difference between the use of fluoride for post-RT dental prevention in the form of 1% sodium fluoride gel versus toothpaste (1350 ppm) to reduce the need of dental extractions due to caries and associated risk of ORN (no ORN cases reported). Two further RCTs focused on the use of HBO. In 1985, Marx et al. [75] showed that HBO decreased ORN onset compared to patients treated with antibiotics following the removal of teeth, while the other study [76] could not identify any difference between the combined use of HBO and antibiotics compared to antibiotics alone. Although most of the literature focused on HBO [72] and autologous platelet concentrates [77] showing controversial findings, other medical approaches have been proposed, including the combined use of pentoxifylline and tocopherol (in the so-called PENTO protocol). Of note, in the majority of studies involving both HBO or PENTO protocol, the additional use of antibiotics was habitually prescribed [72].

The next paragraphs describe the PENTO protocol and HBO as preventive strategies for ORN in case of dental extractions.

#### 3.2.1. Pentoxifylline and Tocopherol

The recent RIF theory supported the use of anti-fibrotic and anti-oxidant agents for the treatment of ORN. Some studies, in particular, investigated the combined use of pentoxifylline in combination with tocopherol, in a specific pharmacological protocol called PENTO (from pentoxifylline “PEN” and tocopherol “TO”) [18,78]. Pentoxifylline is a methylxanthine derivative able to increase vascularization and reduce inflammatory mediators, and it is already in use for the treatment of vascular diseases. Tocopherol is a methylated phenolic compound belonging to the vitamin E group, which acts as an anti-oxidant [78]. The combined use of pentoxifylline and tocopherol counteracts the development of fibrotic and inflammatory bone damage caused by RT, and it represents a low-cost and easy clinical approach [79].

**Table 1:** Implant placement in patients receiving head and neck RT.

PubMedID	First Author	Year	Study Design	No. of Included Studies	No. of Included Patients	Mean Delay RT—Implant Placement	Implant Survival	Other Risk Factors Associated with Reduced Implant Survival	Follow-Up Time	Conclusions/Reccomandations
31612191	Di Carlo, S. [71]	2019	Retrospective study	/	17	14	90.50%	/	>12 m	Better outcomes when the implant was placed at least after 14 months and not loaded until at least 6 months after placement.
34903387	Shokouhi, B. [59]	2022	Systematic review and meta-analysis	7	441	6–18 m	/	RT doses > 50 Gy Implant placed in the maxilla	1–14 y	Implant survival is significantly lower in RT compared with non-RT patients (*p* < 0.001). Implant placement should be delayed by at least six months following RT.
34255187	Schiegnitz, E. [62]	2021	Retrospective study	/	164	43.6 m	87.3% (5 y), 80.0% (10 y) at time of surgery—92.5% (5 y), 89.5 (10 y) after oncological treatment	Implant placed in augmented and irradiated bone	37–49 m	A successful and safe rehabilitation of the irradiated oral cancer patient with high implant survival rates is possible for either secondary or primary placed implants.
33278135	Veld, MI. [80]	2021	Systematic review	10	/	/	90.4–100%	/	12–174 m	Slightly higher survival of immediately placed implants compared with postponed placed implants (*p* = 0.81). RT vs. non-RT showed a better survival of immediately placed implants not having received RT (*p* = 0.10).
31898358	Koudougou, C. [56]	2020	Literature review	4	341	/	82–96.7%	/	29–60 m	The outcomes for implant survival rates appear to be positive for irradiated implants. All mandibular implants were selected for this review.
27034761	Shugaa-Addin, B. [63]	2016	Literature review	18	1175	/	74.4–97%	Maxillary implants RT doses > 70 Gy	0.5–10	Dental implants may be affected by RT, especially when they are placed in the maxilla, in grafted bone, or after radiation; however, they remain a functional option for the rehabilitation of HNC patients.
20701621	Korfage, A. [57]	2010	Prospective study	/	50	Time of surgery	89.40%	/	5y	Oral cancer patients can benefit from implants placed during ablative surgery, with a high survival rate of the implants, a high percentage of rehabilitated patients, and a high denture satisfaction up to 5 years after treatment.
25926008	Zen Filho, EV. [68]	2016	Systematic review	8	331	1–20 m	/	RT doses > 50 Gy	1–168 m	The placement of implants in the irradiated bone is viable, and head and neck RT should not be considered as an absolute contraindication for dental rehabilitation with implants.
23742098	Piardi Claudy, M. [70]	2013	Systematic review and meta-analysis	10	39	/	13.6% (risk of failure)	Placement of dental implants between 6 and 12 months post-RT	1–170 m	Placing implants in the bone within a period shorter than 12 months after RT may result in a higher risk of failure.

m = months, ORN = osteoradionecrosis, RT = radiotherapy, y = years.

**Figure 1:**
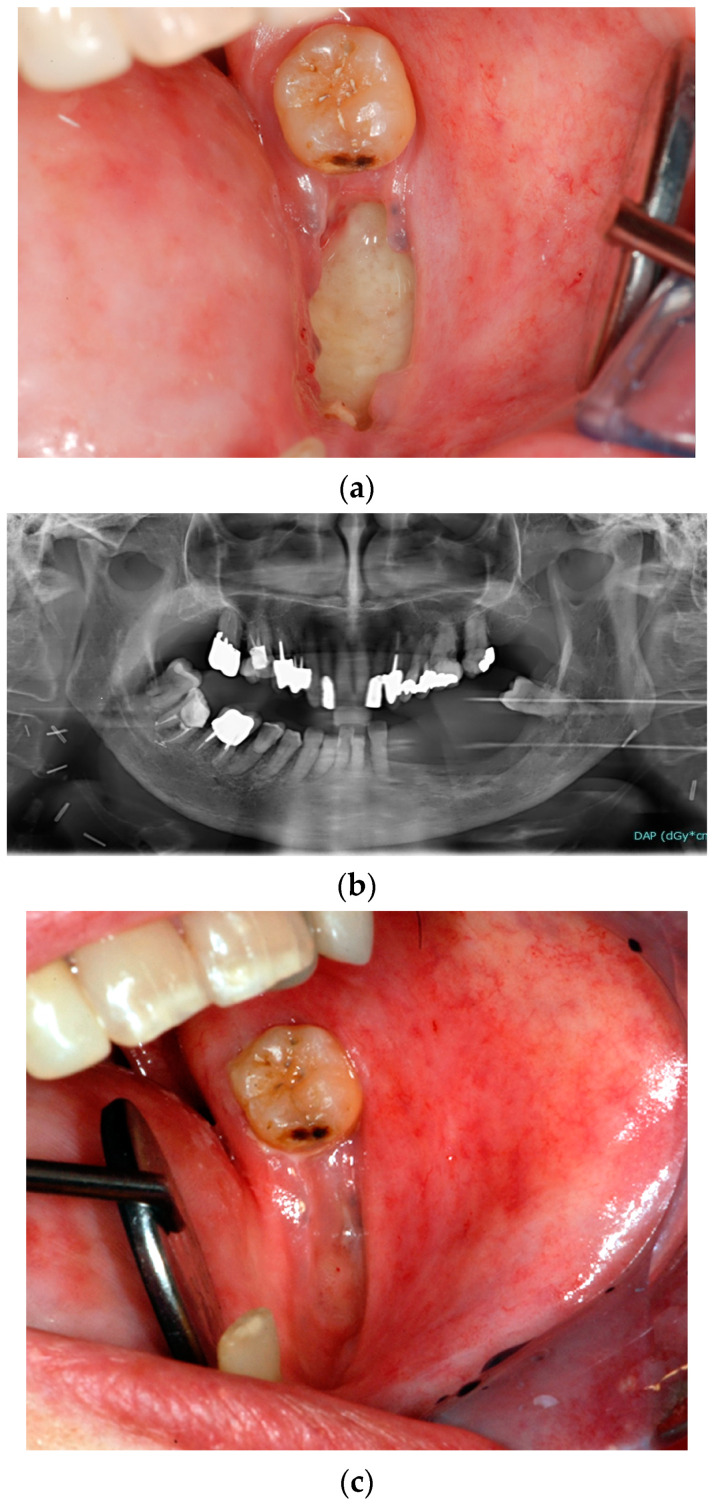
Clinical case of a patient, who received RT for OSCC and developed ORN of the left mandible: clinical picture of the necrotic bone lesion (**a**) and related orthopantomography (**b**). The patient was treated with bone sequestrectomy under PENTO protocol: six months after conservative surgery for removing bone sequestration, the complete healing could be observed (**c**).

The use of the PENTO protocol as a therapeutic regimen has been reported for the management of ORN [81,82] in order to promote bone sequestration until spontaneous bone removal or to facilitate the following surgical procedures of bone sequestrectomy (Figure 1). Similarly, the PENTO protocol has been proposed for the management of medication-related ORN of the jaw (MRONJ) [83].

**Figure 2:**
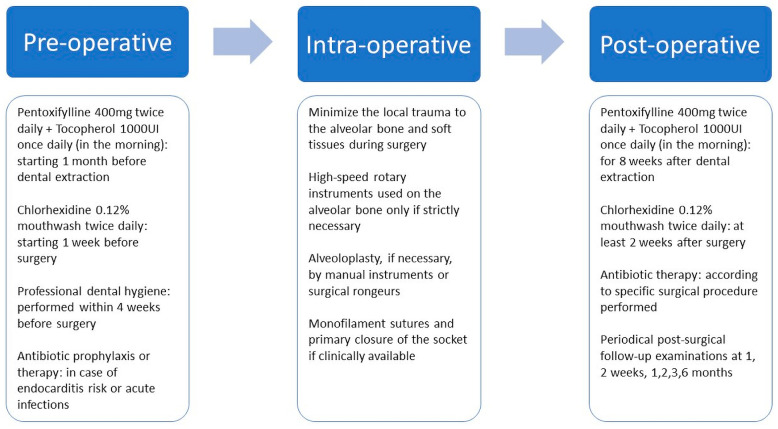
The proposed PENTO prophylactic protocol for the prevention of ORN in patients who underwent oral surgery. The additional use of antibiotics should be prudentially evaluated case by case, considering the clinical picture, the presence of dental infection, the patient-related infective risk, and the surgical procedure.

The PENTO protocol has been proposed as a prophylactic therapy for ORN in patients requiring oral surgery procedures after undergoing head and neck RT, but very few data are available [84]. To the best of our knowledge, only three clinical studies had evaluated the effectiveness of PENTO as a prophylactic therapy for ORN [78,85,86] using slightly different dosages from one study to another. Patel et al. and Aggarwal et al. reported ORN incidences after dental extractions in patients who previously underwent head and neck RT of 1.2% and 1.8%, respectively [85,86]. In both studies, pentoxifylline 400 mg twice daily and tocopherol 1000 IU daily were prescribed starting from 1 month before surgery, then continued after the intervention until complete socket healing [85,86]. Lombardi et al. reported an ORN incidence of 5.6% after the administration of pentoxifylline 400 mg twice a day and tocopherol 800 IU once a day, starting from 1 week before the surgical procedure and continuing 8 weeks after [78]. The PENTO protocol was associated with antibiotics prescription at variable rates among different studies: in 94% of cases in Patel et al., in 100% of cases in Aggarwal et al., while in only 48% of cases in Lombardi et al. [78,85,86]. Gathering the data from the literature, a suggested PENTO protocol for ORN prevention could be proposed, and it is described in Figure 2.

The differences in the ORN rate among the studies on PENTO protocol may be ascribed to the different dosages of pentoxifylline and tocopherol employed and the variable association with antibiotics. Of note, there are different percentages, among studies, of patients receiving head and neck RT for oral cancer: the involvement of the oral cavity in the radiation field is a major risk factor for ORN, and the proportion of OSCC patients was significantly higher in the study by Lombardi et al. (58.7%) over the others [78,85,86].

The main contraindication to PENTO protocol is related to the use of pentoxifylline, which could increase the bleeding in predisposed patients (such as in the case of concomitant oral anticoagulant therapy) [87].

However, at present, no standard of care is currently available for preventing ORN onset following dental extraction, since results concerning the clinical effectiveness of PENTO protocol, despite being interesting, are still scanty and non-homogeneous [84], and further trials will be of relevance to clarify the clinical utility of this approach.

#### 3.2.2. HBO Therapy and ORN Prevention

In irradiated tissues, HBO promotes angiogenesis, enhances neovascularization, fibroblast, and osteoblast proliferation, and increases collagen synthesis, and it has been proposed as both a preventive and therapeutic strategy for ORN [65,88,89,90].

Based on the results of a few outdated studies, antibiotics, local wound care, and/or HBO have been reported to be successful in 25–44% of cases when treating ORN, but the power of these results was weakened by the small sample sizes [11,30,91,92].

A systematic review [47] reported a slight benefit from the use of HBO treatment in irradiated patients after tooth extraction. An ORN incidence of 4% was found when dental extractions were performed under prophylactic HBO versus an incidence of 6% using antibiotics [47]. The increased risk of ORN was also associated with the extraction of mandibular teeth within the radiation field in individuals who received radiation doses greater than 60 Gy [47]. Consistently, the radiation dosage is also correlated with the response to conservative treatment. Most ORN cases, developed after exposure to doses greater than 60 Gy, responded poorly to conservative therapy [11,93].

The recent Cochrane review, mentioned above, was not able to demonstrate with certainty whether or not HBO is effective in avoiding the onset of ORN of the jaw [72]. Only one trial showed that HBO treatment is correlated with a reduction in the development of ORN in comparison with patients treated with antibiotics after dental extractions [75].

At the same time, a randomized controlled phase III trial reported that the incidence of ORN at 6 months after RT was 6.4% and 5.7% for the HBO and control groups, respectively, concluding that, due to the low incidence of ORN, HBO for dental extractions or implant placement in the irradiated mandible was not necessary [94]. Along this line, further studies, including retrospective case series and single institution experiences, showed that the routine use of HBO for the prevention or management of ORN of the jaws and the reduction in the incidence of ORN in irradiated patients requiring tooth extraction is not recommended [95,96].

The type of irradiation, radiation dose, position of the implant (maxillary or mandibular), and use of HBO have been discussed as factors that may affect the survival of dental implants placed in irradiated patients [97,98,99]. According to the survival rates of dental implants reported by the work by Benites Condezo et al., there was no evidence that irradiated patients, who underwent HBO treatment before implant placement as an adjuvant treatment, had a lower failure risk than those who did not [100].

Some limitations regarding the use of HBO should be also considered, including the high number of sessions, low availability of facilities, high costs, and specific contraindications (for example, in the case of lung diseases). Considering these drawbacks and the poor evidence reported in the current literature, HBO therapy might not be routinely recommended for the prevention of ORN as an adjuvant treatment in case of dental implant or dental extraction in irradiated patients [72].

## 4. Study Limitations and Future Perspectives

The current review is a narrative review with the aim of providing updates on ORN in head and neck cancer patients who need oral surgery. Because it is not systematic, it displays intrinsic drawbacks related to potential subjectivity in retrieving, selecting, and interpreting the main findings. However, the overall analysis of the literature, although based only on the PubMed database, can support the need for further studies to better identify all confounding factors affecting ORN development.

Implication for research—The most suitable study design for assessing the risk of ORN and the utility of preventive strategies would be a double-blinded randomized clinical trial that allocates HNC patients to groups in which dental extraction is performed prior to, during, or after RT and determines the frequency of ORN among the groups. However, this study design would be ethically challenging and would require a multicenter design to achieve adequate sample size and long-term follow up. These characteristics may explain the lack of studies addressing dental extractions in the pre-RT setting. Future directions of research, in particular, should provide details on the type of HNC cancers, especially oral cancers, which predispose the patient to higher ORN risk, the oral surgery protocol, and the type and dosage of RT received. Machine learning methods for the prediction of ORN incidence are expected to be further explored in the attempt to support clinicians in stratifying the risk. Regarding ORN prevention, a standardized protocol for administering the PENTO therapy should be better defined, clarifying the pharmacological doses and the role of additional antibiotics. On what concerns HBO therapy, future studies should include an economic evaluation, considering to apply this approach to selected patients. Finally, the increasing use of proton therapy requires a better understanding of the long-term side effects.

Implication for clinical practice—Overall, an established standard of care and high-quality evidence for ORN prevention in the case of dental extraction and dental implant placement are lacking. Some studies, which investigated ORN risk factors and the efficacy of antibiotics, HBO, and the combination of pentoxifylline and tocopherol (PENTO protocol), did not reach conclusive assertions. There is an urgent need to improve research for more efficacious clinical decision making. In the future, the achievement of a general evidence-based consensus in patient management will be of pivotal help for dental clinicians.

## 5. Conclusions

The risk of ORN remains persistent throughout life, and there is no general consensus on the correct preventive management of irradiated HNC patients who receive dento-alveolar surgery.

Early pre-RT involvement of dental specialists and oral and maxillofacial surgeons is crucial for proper dental care. Current guidelines recommend a complete dental check-up before the RT, with tooth extractions at least 7–14 days prior to RT to reduce the risk of ORN.

After RT, traditional removable prostheses should be the first choice, while great caution is advised in placing dental implants due to anatomical complexities and the lifelong ORN risk. Implant placement should be reserved only for highly selected cases under informed, patient-specific consent, after consultation with a radiation oncologist.

There is no consensus on the best timeframe between the end of the RT and the dento-alveolar surgery to reduce the risk of ORN; some preventive strategies have been proposed and include the use of antibiotics, HBO, and the combination of pentoxifylline and tocopherol (PENTO protocol), but, to date, an established standard of care is lacking.

Further studies are needed to further clarify risk factors for ORN onset and to assess the efficacy of preventive strategies.

## Data Availability

No new data were created or analyzed in this study. Data sharing is not applicable to this article. Supplementary Materials summarize the main articles considered for the review.

## Supplementary Materials

Supplementary materials include the summary tables of the retrieved articles, reporting their main findings and details. The following supporting information can be downloaded at https://www.mdpi.com/article/10.3390/biomedicines11123339/s1, Table S1: Timing for dental extractions: pre-RT; Table S2: Timing for dental extractions: after RT.

## Abbreviations

HBO	hyperbaric oxygen
HNC	head and neck cancer
HPV	human papillomavirus
IMRT	intensity-modulated RT
MRONJ	medication-related ORN of the jaw
ORN	osteoradionecrosis
PENTO	pentoxifylline and tocopherol
RIF	radiation-induced fibrosis
RT	radiation therapy
3DCRT	three-dimensional conformal RT
