# Supplementary material for: Oral Surgery and Osteoradionecrosis in Patients Undergoing Head and Neck Radiation Therapy: An Update of the Current Literature
Source: Biomedicines. 2023 Dec 18;11(12):3339. doi: 10.3390/biomedicines11123339 (PMC10742198; doi:10.3390/biomedicines11123339)
Supplement: Supplementary file 1 [file biomedicines-11-03339-s001.zip › biomedicines-2713338-supplementary.pdf]

Supplementary Information

Table S1: Timing for dental extractions: pre-RT

PubMedID	First Author	Year	Study design	Nr of included studies	Nr of included patients	% ORN	Range RT-ORN	Site of ORN	Associated risk factors	Time between extraction and RT	follow-up time	Conclusions/Reccomandations
32531873	Lajolo C	2021	Systematic review	8	494	2.2	/	Mandible	No	Only 13 studies: mean 24,7 d	30.6 m	Teeth extractions before RT represent a risk factor for ORN, even it is generally recommended to remove oral foci before RT
35713725	Costa Normando AG	2022	Systematic review and meta anaysis	28	33662	7	0-30 y	Mandible (N=804) Maxilla (N=64)	/	14-34 m	0–34 y	27/28 studies demonstrated a significant association between an increased risk of ORN and post-RT tooth extraction (odds ratio: 1.98; 95% CI: 1.17–3.35; p=0.01)
35246621	Ward R	2022	Retrospective study	/	154	1.3	/	Mandible	/	10-21 d	> 20 m	No apparent correlation with healing time between extractions and RT
35031745	King R	2022	Prospective study	/	145	0	/	/	/	13 d	< 24 m	OPGs should be part of initial HNC staging and referral to Regional Oncology Centre dental services should be made as part of the pre-RT workup

33685773	Beaumont S	2020	Literature review	21	14389/36294	5.50	/	/	/	/	6 m- 17 y	There was no statistically significant difference between extractions performed prior or after RT
28862391	See Toh YL	2017	Retrospective study	/	207/231	2.60	1-24 m	Mandible and maxilla	Smoking and increased number of teeth removed	/	52 m	No correlation between dental extractions pre- or post-RT and the development of ORN
27473832	Beech NM	2017	Retrospective study	/	129/190	19	/	Mandible (N=26) Maxilla (N=3)	Smoking status, p16 status negative	/	/	Pre-RT dental extractions appear to not protect against the development of ORN
35029717	Balermpas P	2021	Systematic review and meta-analysis	7	432/875	21	1-67.7 m	Mandible and maxilla	/	/	/	Tooth extraction before IMRT is more common than after IMRT, but dental extractions before compared to extractions after IMRT have not been proven to reduce the incidence of ORN. Pre-RT dental care and extractions remain the standard procedure to prevent dental complications from IMRT.

HNC= Head and Neck cancer, IMRT= intensity modulated RT, m= months, OPG= orthopantomography, ORN=osteoradionecrosis, RT= Radiotherapy, y=years

Table S2: Timing for dental extractions: after RT

PubMedID	First Author	Year	Study design	Nr of included studies	Nr of included patients	% ORN	Range RT-ORN	Site of ORN	Associated risk factors	Time between extraction and RT	follow-up time	Conclusions/Reccomandations
35713725	Costa Normando AG	2022	Systematic review and meta anaysis	28	11705	11	0-30	Mandible (N=804) Maxilla (N=64)	/	/	0–34 m	27/28 studies demonstrated a significant association between an increased risk of ORN and post-RT tooth extraction (odds ratio: 1.98; 95% CI: 1.17–3.35; p=0.01)
33685773	Beaumont S	2020	Literature review	21	6030/37805	5.30	/	/	/	> 6 mesi	6 m- 17 y	There was no statistically significant difference between extractions performed prior or after RT
27401528	Wanifuchi S	2016	Retrospective study	/	12206	100	/	Mandible, molar region	association between the irradiation field and the site of ORN development	37.5 m	/	All patients who received tooth extraction after RT developed ORN (100 %) independently of time between tooth extraction and the end of RT
28862391	See Toh YL	2017	Retrospective study	/	16/231	0	1-24 m	Mandible and maxilla	Smoking and increased number of teeth removed	/	52 m	No correlation between dental extractions pre- or post-RT and the development of ORN

27086489	Kuo T	2016	Retrospective study	/	522/1759	2.22	3.02 y	Mandible	number of teeth removed, > timing of extraction after RT	stratified if > or < 6 m	3.65 y	A tooth extraction time less than half a year after RT or during the head and neck RT period, and extraction tooth number ⩽5 would significant lower the ORN prevalence.
21115324	Nabil S	2011	Systematic Review	19	828	7	/	Mandible and maxilla	protective factors: prophylactics HBO and antibiotics - risk factor: RT dose> 60Gy	/	1-42 m	Incidence of ORN after post-RT tooth extractions is low, the extraction of mandibular teeth within the radiation field in patients who received a RT> 60 Gy represents the highest risk of developing ORN
34261515	Khoo SC	2021	Retrospective study	/	73	21.9	/	Mandible and maxilla	Extraction > 5 y after RT, surgical removal procedure and invisible upper cortical line of mandibular canal	mean year of 9.02±6.57 y	/	Extraction more than 5 years after radiotherapy, surgical removal procedure and invisible upper cortical line of mandibular canal on the DPT were the predictors of ORN
28370713	Wang T	2017	Retrospective study	/	4513/23527	3.93 per 100 person-	222 d	Mandible and maxilla	Protective effect: steroids	3 y	43.778 person-years	Post-RT extraction was associated with gradually increased risk of ORNJ over time that peaked at 4 to 5 years

						years						
17222536	Lye KW	2007	Prospective study	/	40	1.90	/	Mandible and maxilla	/	5.6 y	/	Post-RT extractions have a low risk of complications and the results point to age as a factor that may influence wound healing

DPT =dental panoramic tomogram, m= months, ORN=osteoradionecrosis, RT= Radiotherapy, y=years
